# SnoN Facilitates Axonal Regeneration after Spinal Cord Injury

**DOI:** 10.1371/journal.pone.0071906

**Published:** 2013-08-02

**Authors:** Jiun L. Do, Azad Bonni, Mark H. Tuszynski

**Affiliations:** 1 Department of Neurosciences, University of California San Diego, La Jolla, California, United States of America; 2 Department of Pathology, Harvard Medical School, Boston, Massachusetts, United States of America; 3 Veterans Affairs Medical Center, San Diego, California, United States of America; Hertie Institute for Clinical Brain Research, University of Tuebingen., Germany

## Abstract

Adult CNS neurons exhibit a reduced capacity for growth compared to developing neurons, due in part to downregulation of growth-associated genes as development is completed. We tested the hypothesis that SnoN, an embryonically regulated transcription factor that specifies growth of the *axonal* compartment, can enhance growth in injured adult neurons. *In vitro*, SnoN overexpression in dissociated adult DRG neuronal cultures significantly enhanced neurite outgrowth. Moreover, TGF-β1, a negative regulator of SnoN, inhibited neurite outgrowth, and SnoN over-expression overcame this inhibition. We then examined whether SnoN influenced axonal regeneration *in vivo*: indeed, expression of a mutant form of SnoN resistant to degradation significantly enhanced axonal regeneration following cervical spinal cord injury, despite peri-lesional upregulation of TGF-β1. Thus, a developmental mechanism that specifies extension of the axonal compartment also promotes axonal regeneration after adult CNS injury.

## Introduction

Several mechanisms contribute to the failure of axonal regeneration in the adult central nervous system, including factors both intrinsic and extrinsic to the neuron. The importance of neuron-intrinsic mechanisms in regeneration failure are illustrated by the effects of a “conditioning” lesion, wherein injury to the peripheral process of a dorsal root ganglion (DRG) neuron substantially enhances regeneration of the central process of the same neuron after spinal cord injury [1,2], mediated in part by upregulation of neuronal regeneration-associated genes [3–5]. However, the full set of intrinsic neuronal mechanisms that underlie central axonal regeneration failure remain poorly understood.

Studies of neural development may provide insight into neuron-intrinsic mechanisms influencing axonal growth, since developing axons generally exhibit greater growth capacity than mature neurons [6,7]. SnoN is of particular interest in this respect: SnoN is a developmentally regulated gene that specifies growth of the developing axonal compartment [8]. Overexpression of SnoN itself, or a mutant form of SnoN resistant to ubiquitination and degradation, leads to enhancement of growth in developing neurons specifically in the axonal compartment [8]. Moreover, SnoN-mediated enhancement of axonal growth during development appears to be terminated by transforming growth factor-beta (TGF-β), which facilitates SnoN ubiquitination and degradation through induction of SMAD2 phosphorylation [9].

Accordingly, we examined alterations in these signaling systems in adult neurons in vitro and after spinal cord injury in vivo. We now find that enhancement of SnoN signaling significantly enhances growth from adult dorsal root ganglion neurons in vitro, and significantly enhances axon regeneration after spinal cord injury.

## Materials and Methods

The VA Institutional Animal Care and Use Committee approved this research. National Institutes of Health and Institutional Animal Use and Safety Committee guidelines for laboratory animal care and safety were strictly followed for all animal use and post-operative care.

### Experimental Design

We first examined effects of SnoN and its negative modulator, TGFβ1, on neurite outgrowth in cultured adult DRG neurons. Next, we examined whether expression of a mutated form of SnoN that is resistant to degradation enhanced axonal regeneration after C3 spinal cord injury.

### Expression Constructs

We generated dual promoter expression vectors that expressed copepod green fluorescent protein (copGFP) as a reporter, and either: 1) the 2055 bp cDNA for SnoN; 2) a mutant, ubiquitination/degradation-resistant form of SnoN containing a D-Box mutation (SnoN-DBM) in its base pair coding region of 490-501 [10]; or 3) a control vector expressing the reporter gene GFP (i.e., a GFP – copGFP vector). GFP, GFP-SnoN, or GFP-SnoN-D-box mutation plasmids were expressed by a hybrid chick beta actin – minimal CMV (CAG) promoter, and the reporter gene copGFP was driven by an EF1-α promoter, as previously described [11]. To facilitate histological detection of SnoN, we fused the GFP reporter to the amino terminal of SnoN; the resulting fusion protein was functional, as shown in Results. Vectors were cloned into an adeno-associated virus serotype 6 backbone (AAV6), as previously described [11].

For in vivo studies, we generated single-stranded recombinant AAV-serotype 6 viral vectors containing the human cytomegalovirus promoter, SV40 intron, enhanced GFP or SnoN-DBM transgene, and a SV40 poly adenylation signal. Vector production was performed by the Vector Core at the University of North Carolina using methods previously described [12]. Briefly, HEK293 cells were transfected with the appropriate plasmids by CaCl_2_ transfection and resultant viral particles were purified over CsCl density gradients. Dot-blot DNA hybridization was used to determine viral titers. Prior to surgery, viral stocks were diluted to 1.0x10^12^ infectious units/ml in Hanks’ balanced salt solution.

### Adult DRG Neuronal Culture

DRGs were dissected from adult female Fisher 344 rats weighing 150-165 g, digested in 0.25% collagenase type XI and 5µg/ml dispase, and triturated. For transfections, 1 million cells and 10 µg of DNA were used according to Nucleofactor (Lonza) protocols. Briefly, cells were centrifuged and resuspended in Rat Neuron Nucleofector solution. Cell suspensions were mixed with DNA and transferred to a cuvette. Suspensions were electroporated using Nucleofector Program G-013 and seeded at low density on cell culture plates (Corning #3516) coated with poly-L-lysine (20µg/mL) and laminin (0.5µg/mL; this is a low dose of laminin that facilitates detection of enhanced outgrowth after administration of test substances). Cells were cultured in media (DMEM: F12 supplemented with B27, 100U/mL penicillin-streptomycin, and 2mM glutamine, Invitrogen) supplemented with TGF-β1 (100 ng/ml). Cells were fixed with 4% PFA after two days in vitro and labeled for βIII-tubulin (@1:1000 concentration; Promega G712A), NF200 (@1:6000 concentration; Chemicon MAB5262), and copGFP (@1:5000 concentration; Evrogen AB502). Images were acquired using an ImageXpress Micro system (Molecular Devices) and analyzed using the neurite outgrowth module of the MetaXpress software package. For quantification of transfected neurons, only neurons expressing the copGFP reporter were analyzed. The program software traces the processes of all objects with neuronal morphology, and in an automated fashion provides mean neurite length and longest neurite from each cell. Optimization of the software was performed prior to use in this experiment to ensure that object identification by the automated system, and neurite quantification, were consistent with manual methods performed on sample photographed images by human observers. Cells were plated at sufficiently low densities to permit identification of individual neurons and their processes in more than 95% of sampled neurons. All experiments were repeated in triplicate and a minimum of 150 neurons per condition were quantified.

### Protein Isolation and Western Blot Analysis

Protein was extracted from DRG cultures or from adult L4 and L5 DRGs using RIPA buffer. Protein concentration was determined using Bio-Rad Protein Assay and 20 µg was resolved on a 4-12% SDS-PAGE gel (Invitrogen). The gel was transferred to 0.45 µm PVDF membranes (Millipore), blocked with 5% nonfat milk, washed with 0.05% Tween-20 in PBS, and incubated with anti-pSMAD2 (@1:1000 concentration; Cell Signaling #3101), anti-SMAD2 (@1:1000 concentration; Cell Signaling #3103), or anti-SnoN (@1:1000 concentration; Cell Signaling #4973). Blots were washed and incubated with horseradish peroxidase conjugated secondary antibodies and visualized and analyzed using chemiluminescence on a Bio-Rad GelDoc. Blots were then stripped and reblotted with anti-β-actin (@1:1000; Cell Signaling #3700) for loading controls.

### Surgery, Tissue Processing and Axon Quantification

Adult female Fisher 344 rats weighing 150-165 g were anesthetized with a mixture (2 ml/kg) of ketamine (25 mg/ml), rompun (1.8 mg/ml) and acepromazine (0.25 mg/ml). National Institutes of Health and Institutional Animal Use and Safety Committee guidelines for laboratory animal care and safety were strictly followed for all animal use and post-operative care. For intraganglionic injections, the vertebrate overlaying the L4 and L5 DRGs were removed and 1 µl per DRG of AAV6 (1.0x10^12^ infectious units/ml) was injected into the L4 and L5 DRGs bilaterally using a pulled glass micropipette and picospritzer. Great care was taken to avoid damage to the injected DRG as this might “condition” the neurons. One week later, C3 dorsal column lesions were performed using a tungsten wire knife in 27 animals. The lesion site was filled with syngeneic bone marrow stromal cells to provide a matrix in the lesion site to which injured axons could attach and grow [1,13]; without such a matrix, axons are unable to regenerate into the lesion. Rat marrow stromal cells were accordingly prepared [1,14] and transplanted into the lesion site using glass pipettes and a picospritzer; 2 µl were injected at a concentration of 50,000 cells/µl over one minute. 12 animals received lesions and injections of SnoN-DBM into the L4/5 DRGs, and 11 control-lesioned animals received injections of AAV6-GFP into the L4/5 DRGS. Four additional controls received lesions and cells only without vector injections. Four weeks after lesions and three days prior to perfusion, dorsal column sensory axons were labeled transganglionically by cholera toxin B subunit (CTB) injection into the sciatic nerve (2 µl of 1% solution per sciatic nerve) as described previously [1,15–17]. Animals were then transcardially perfused with 4% paraformaldehyde, post-fixed overnight, and cryoprotected in 30% sucrose at 4° C.

For immunolabeling of DRGs, L4 and L5 DRGs were sectioned on a cryostat set at 10µm thickness, directly mounted on microscope slides, and thoroughly dried. Sections were washed with tris-buffered saline and blocked with 5% goat serum for 1 hr at room temperature. Sections were incubated overnight at 4° C with anti-GFP (@1:1000; Invitrogen A6455), anti-SnoN (@1:1000 concentration; Santa Cruz SC-9141), or βIII-tubulin (@1:1000 concentration; Cell Signaling #2128). After washing, sections were incubated with Alexa 488 and/or Alexa 594 secondary antibodies for 2.5 hr at room temperature.

Spinal cords were sectioned sagittally on a cryostat set to 30 µm intervals. All sections were processed free-floating. For visualization of CTB-labeled sensory axons, endogenous peroxidase activity was blocked with 0.6% hydrogen peroxide and non-specific antibody reactions were blocked with 5% horse serum for 1 hr at room temperature. Sections were incubated for 72 hr at 4° C with the primary CTB antibody followed by incubation with a biotinylated horse anti-goat IgG secondary antibody for 1 hr at room temperature. After 1 hr incubation in avidin-biotin peroxidase complex at room temperature, diaminobenzidine (0.05%) with nickel chloride (0.04%) were used as chromogens. Glial fibrillary acidic protein (GFAP) was detected subsequently in the same sections by fluorescence labeling using anti-GFAP (Chemicon MAB360) incubated overnight at 4° C. After washes, sections were incubated with Alexa 594 fluorophore-conjugated secondary antibody for 2.5 hr at room temperature. To quantify the mean number of dorsal column sensory axons penetrating the lesion site, a series of 1-in-6 sections was labeled for CTB and GFAP, and the number of CTB-labeled axons present within the lesion site was counted using a 10x ocular with a calibrated grid and a 40x objective. Multiple axons that could not be resolved as separate axons were quantified as a single axon. The observer was blinded to group identity. To compensate for individual differences in efficiency of axon tracing between subjects, the total number of dorsal column sensory axons intersecting a line drawn 500µm caudal to the lesion border was also quantified. The mean number of axons penetrating the lesion site was expressed as a proportion of total axons labeled per subject. Results were expressed as mean ± SEM.

### Statistical Analysis

Differences among multiple treatment groups were assessed using one-way ANOVA with a significance level of p<0.05; individual group differences were assessed using *post-hoc* Fischer’s. Comparison of two groups was made using two-tailed Student’s t-test with a significance criterion of p<0.05. Data are presented as mean ± standard error of the mean. All analyses were performed in a blinded manner.

## Results

### SnoN Promotes Neurite Outgrowth from Adult DRG Neurons *In Vitro* and Overcomes TGF-β1-Related Inhibition

**Figure 1:**
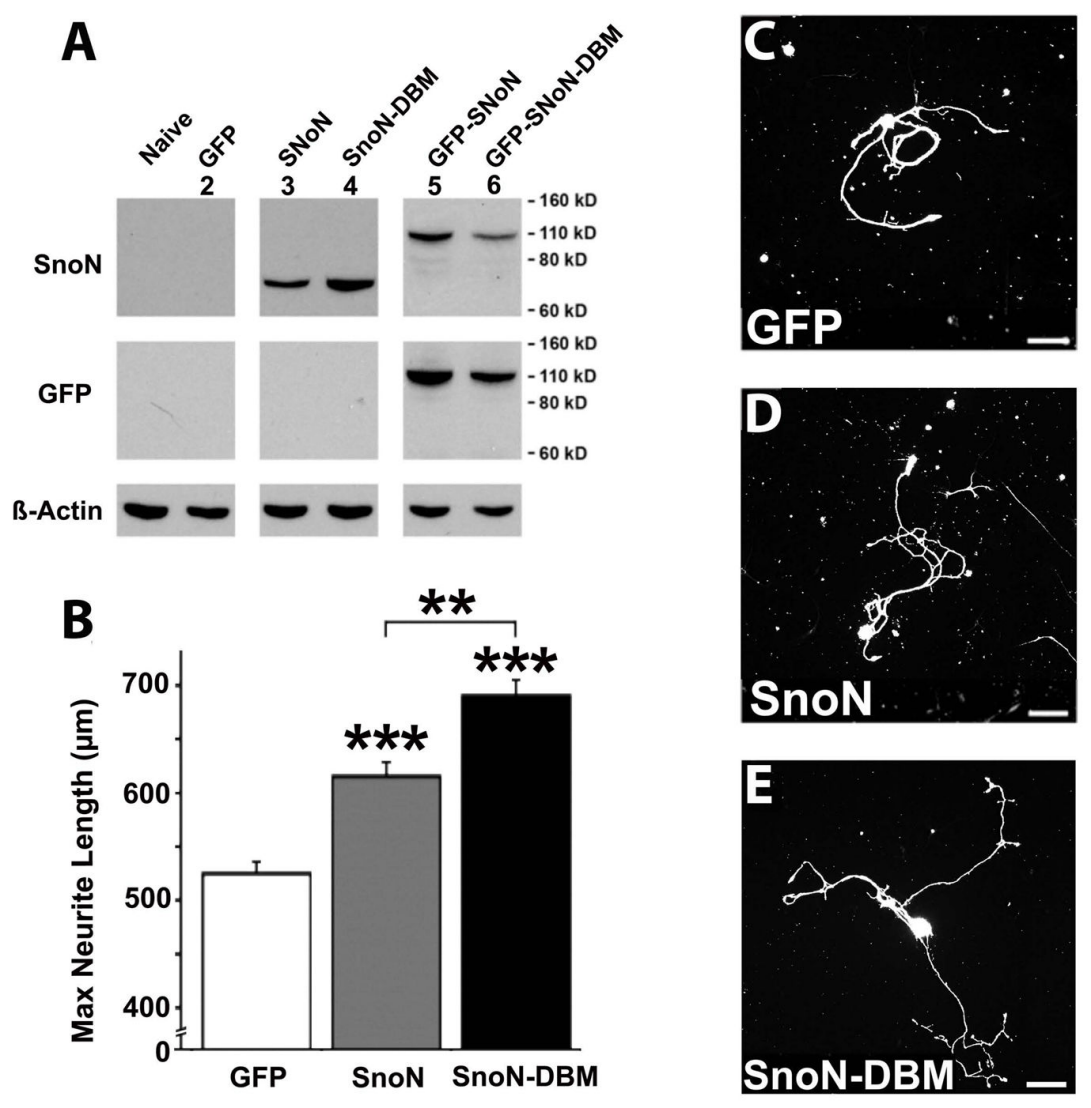
SnoN Promotes Neurite Outgrowth From Adult DRG Cultures. (**A**) Western blot from HEK293 cells electroporated to express GFP, wild-type SnoN, SnoN-DBM, GFP fused SnoN, and GFP fused SnoN-DBM. Cells transfected with wild-type SnoN and SnoN-DBM demonstrate SnoN immunoreactive bands at ~75 kD (lane 3 and 4). Cells transfected with GFP-SnoN and GFP-SnoN-DBM demonstrate SnoN immunoreactive bands and corresponding GFP immunoreactive bands at ~110 kD, a molecular weight consistent with expression a GFP fused variant. (**B**) Neurite outgrowth from control adult DRG neurons in vitro. NF200 label. (**C**) Overexpression of SnoN increases neurite length, quantified in E. Infected cells express GFP and are labeled for NF200 (shown). (**D**) Overexpression of SnoN-D-box mutation further amplifies neurite growth. (**E**) Quantification confirms that SnoN expression in adult DRG neurons significantly enhances neurite growth (ANOVA p<0.001; post hoc Fisher’s ***p<0.001 comparing treated groups to GFP, and **p<0.01 comparing SnoN to SnoN-DBM group).

During neural development, SnoN specifies elongation of the developing axon [8–10]. Accordingly, we determined whether overexpression of SnoN, or expression of a mutant form of SnoN resistant to ubiquitination and degradation (SnoN with a D-Box-Mutation, or SnoN-DBM), enhanced neurite growth from adult DRGs. The SnoN and SnoN-DBM plasmids expressed both SnoN and the reporter gene GFP of the predicted size (Figure 1A). Notably, overexpression of both SnoN and SnoN-DBM resulted in significant increases in neurite outgrowth compared to control neurons after 48 hr *in vitro* (ANOVA p<0.001; post-hoc Fischer’s p<0.001 comparing both conditions to control cells; Figure 1B–E).

**Figure 2:**
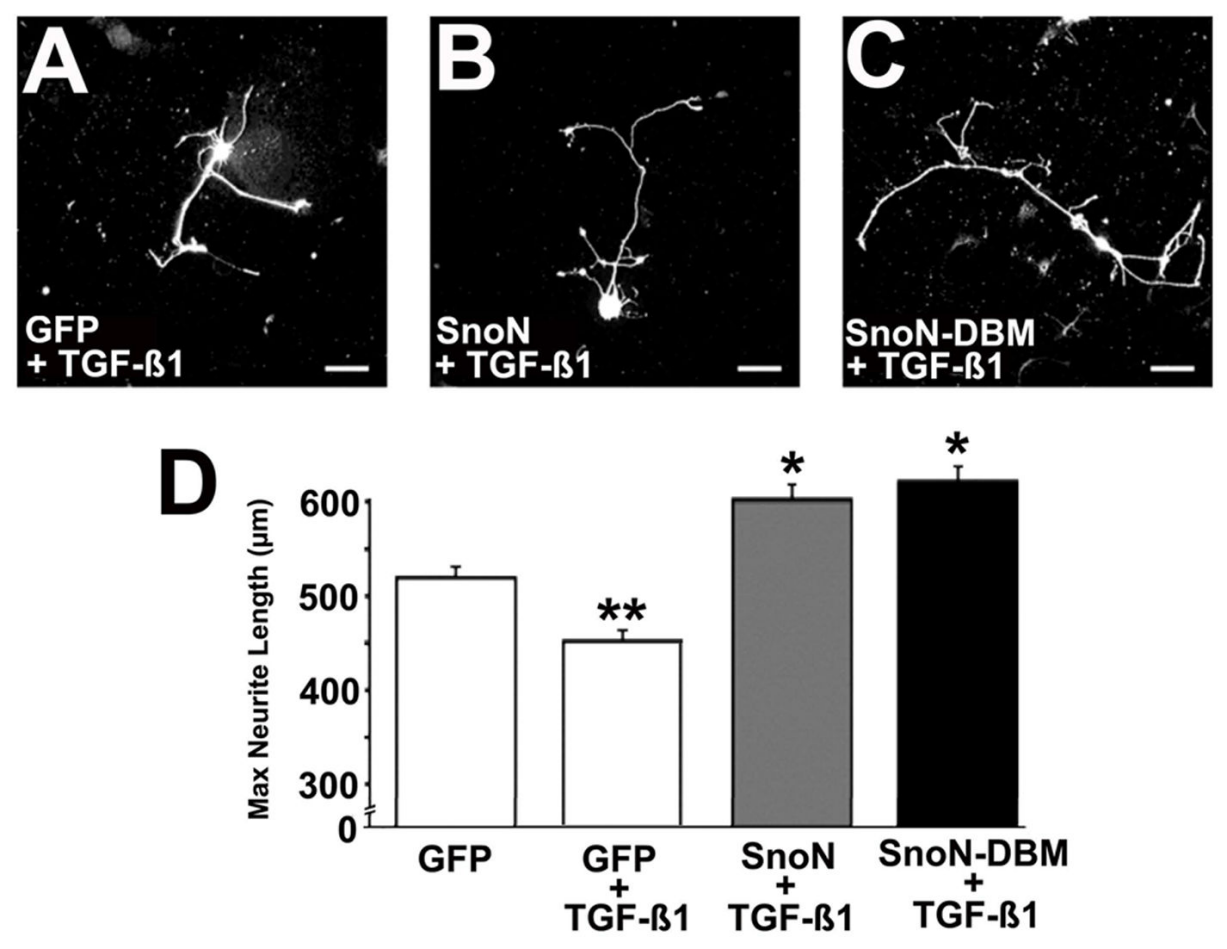
SnoN Overexpression Overcomes TGF-β1 Inhibition. (**A**) Neurite outgrowth in primary adult DRG neurons in the presence of TGF-β1. Cells expressing GFP and NF200 (shown) are quantified. (**B**) Overexpression of SnoN in neurons appears to increase neurite length even in presence of TGF-β1, quantified in D. (**C**) Overexpression of SnoN-D-box mutation also appears to increase neurite growth. (**D**) Quantification reveals that overexpression of TGF-β1 overcomes TGFß-1-mediated inhibition (ANOVA p<0.001; post hoc Fisher’s *p<0.05, **p<0.01).

TGF-β1 acts as a negative regulator of SnoN and is associated with a reduction in axonal growth capacity during neural development [8–10]. We examined whether TGF-β1 also inhibits neurite outgrowth from adult DRG neurons, and whether overexpression of SnoN or SnoN-DMB could overcome inhibition. TGF-β1 (100 ng/ml) significantly reduced neurite outgrowth compared to GFP-expressing control cells (ANOVA p<0.001; post-hoc Fischer’s p<0.05 comparing GFP alone to GFP + TGF-ß1; Figure 2A, D). Overexpression of SnoN or SnoN-DBM in the presence of TGF-β1 significantly increased neurite outgrowth compared to cultures in the presence of TGF-β1 (ANOVA p<0.05, post-hoc Fischer’s p<0.01; Figure 2B–D). Moreover, maximum neurite length also exceeded findings compared to control DRG neurons that lacked TGF-β1 exposure (p<0.05, post-hoc Fischer’s; Figure 2D). Given these collective findings that SnoN can promote neurite outgrowth and overcome TGF-β1-related neurite inhibition, we proceeded to *in vivo* studies in models of spinal cord injury.

### SnoN Promotes Axonal Regeneration After Spinal Cord Injury

**Figure 3:**
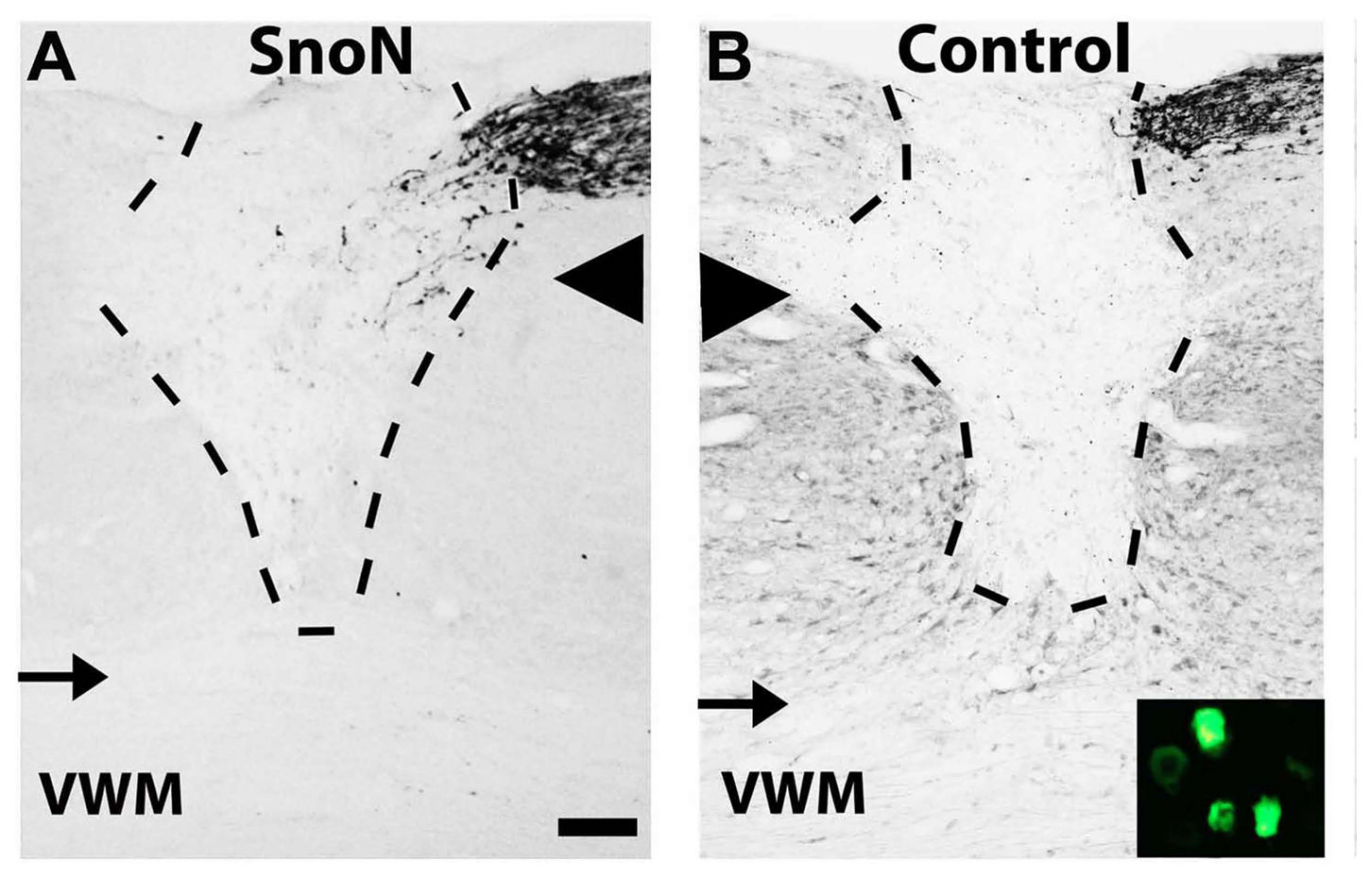
Effects of SnoN Expression on Axonal Regeneration After Spinal Cord Injury. Sensory axonal regeneration was assessed in rats that underwent C3 spinal cord dorsal column lesions. Overview of lesion/graft site in sagittal sections; caudal is to the right and rostral is to the left. CTB-labeled dorsal column sensory axons approach the lesion site from the caudal aspect of the section in panels **A** and **B**. Dashed lines indicate the lesion margins; arrowhead indicates the interface between dorsal white matter and central gray; thin arrow indicates the interface between central gray and ventral white matter (VWM). Inset shows GFP labeling in L4 DRG neurons, indicating persistent gene expression through the experimental period. (**A**) In a lesioned animal that received SnoN-DBM, the dorsal column sensory projection is seen in the upper right hand corner of the section with CTB labeling, as it approaches the lesion site which has been filled with bone marrow stromal cells to provide a permissive matrix for axonal growth. Axons enter the lesion site, visible even at this low magnification. Higher magnification in Figure 5. Dashed lines indicate margins of lesion. (**B**) In a control animal, few axons penetrate the lesion site, more clearly evident in Figure 5. Scale bar A, B: 400 µm.

**Figure 4:**
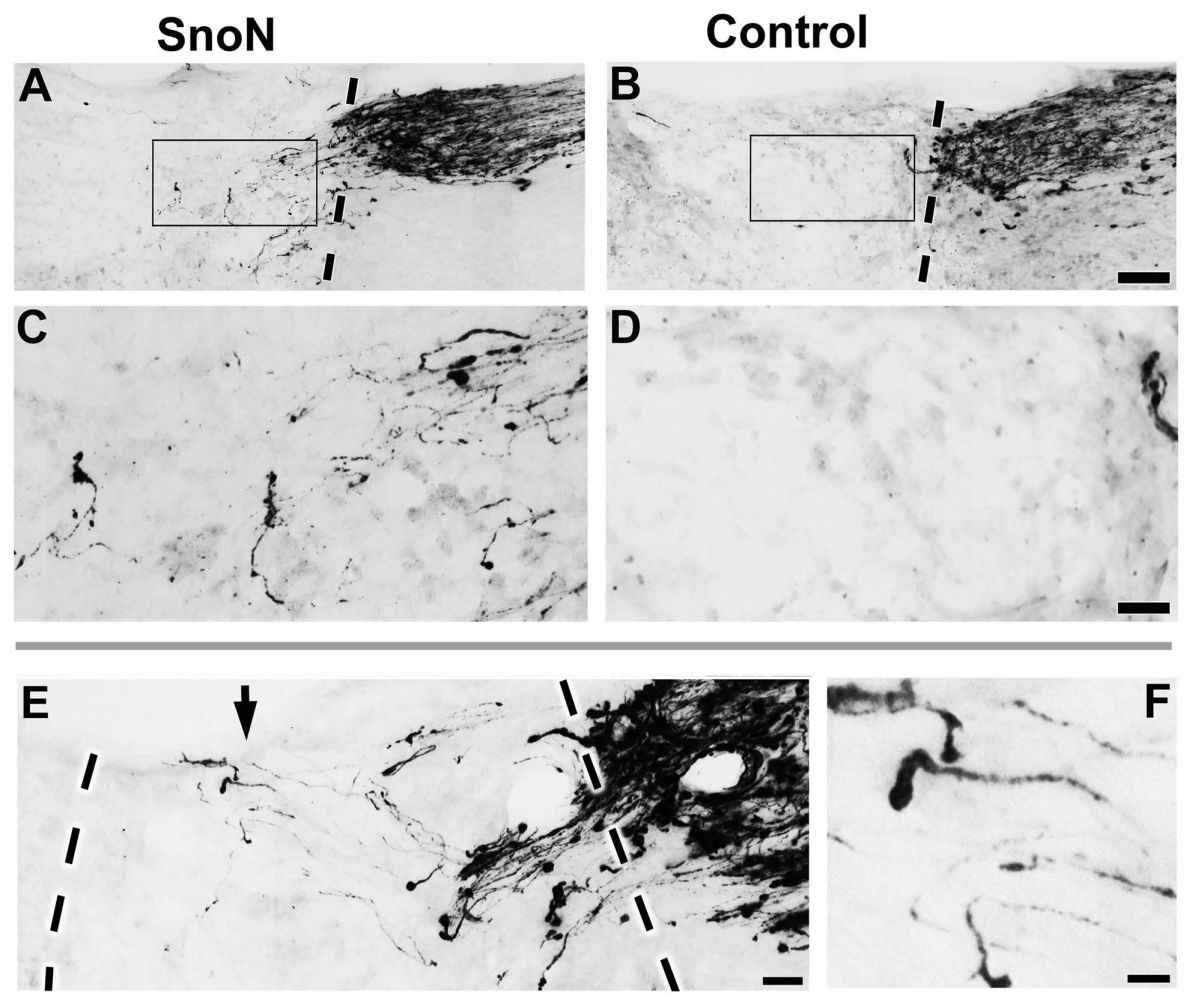
SnoN Promotes Axonal Regeneration After Spinal Cord Injury. Sensory axonal regeneration into site of C3 dorsal column lesion. (**A**, **C**) Animal treated with SnoN-DBM exhibits regeneration into lesion site. Boxed region is magnified in **C**, a region entirely with the lesion site. Axons exhibit circuitous trajectories and make abrupt turns, consistent with regenerating axons (Tuszynski, 2012). Dashed lines indicate the caudal lesion/host interface. (**B**, **D**) Control subjects exhibit few axons within lesion site. Boxed region is entirely within the lesion site and is shown in **D**. (**E**) Another animal that received SnoN-DBM. Several axons are present in the central-distal aspect of lesion site, shown at higher magnification in **F** (arrow). Lesion margins are indicated by dashed lines. Significantly greater numbers of axons penetrate SnoN-DBM treated animals (P < 0.005; see text). Scale bar A, B: 250 µm; C, D: 100 µm; E, 175 µm; F, 50 µm.

**Figure 5:**
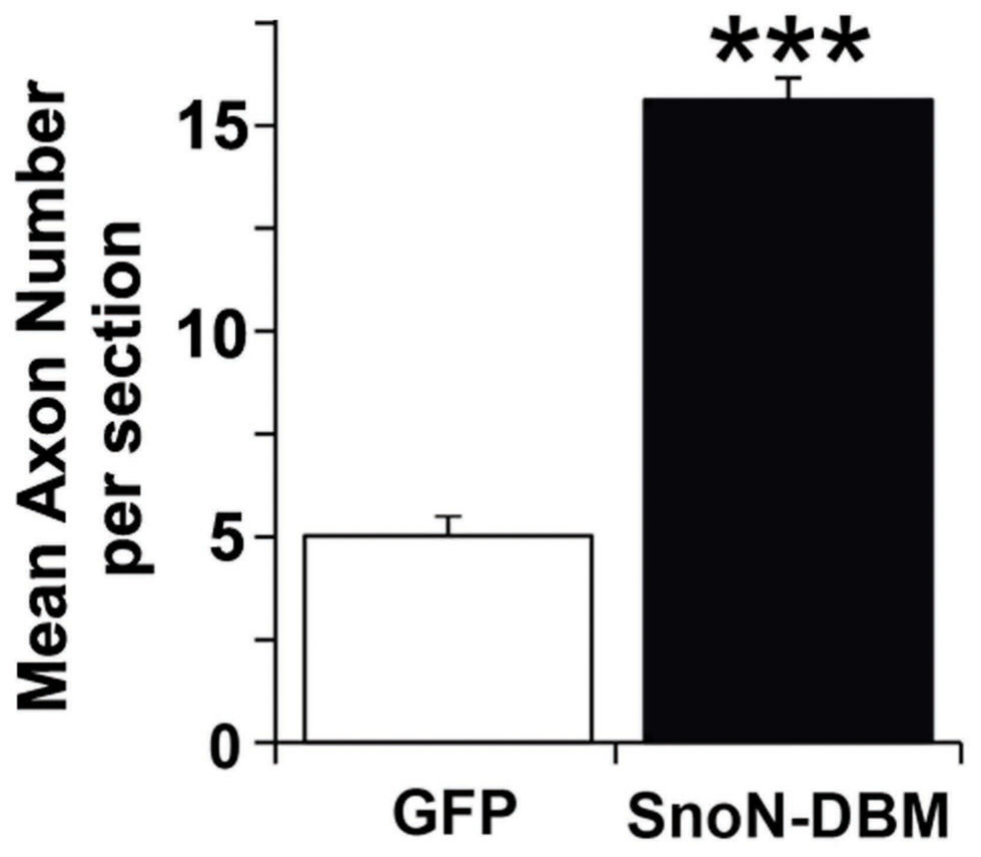
Quantification of SnoN-Induced Axonal Regeneration. Quantification reveals a 3-fold increase in the number of axons regenerating into the lesion site among animals treated with SnoN-DBM. * p<0.001.

For in vivo studies of spinal cord injury, we used the degradation-resistant D-box mutant form of SnoN because of its greater effect in the adult DRG neurite outgrowth assay (Figure 1). AAV6 vectors expressing either GFP or SnoN-DBM were injected into the L4 and L5 dorsal root ganglia using cautious surgical technique and minimal tissue disruption to avoid a traumatic injection-related “conditioning” effect. Gene expression persisted throughout the experimental period, reflected by GFP labeling (Figure 3B, inset). C3 dorsal column lesions were placed, and the lesion site in both GFP and SnoN-DBM groups was filled with marrow stromal cells to provide a permissive matrix for axon growth; without a matrix, axons cannot penetrate the cystic lesion site [1,13]. Notably, four weeks after lesions, a significant, 3-fold increase in the total number of CTB-labeled dorsal column sensory axons regenerating into the lesion site was observed in animals that received infusions of SnoN-DBM compared to controls (p<0.001; Figures 3-5). Moreover, when compared to the total number of CTB-labeled axons in the dorsal column tract approaching the lesion, 31% of all sensory axons in SnoN-DBM-treated animals regenerated into the lesion site, compared to only 7.7% in controls, a 4-fold increase. There was no significant difference in the efficiency of axonal labeling in the control and SnoN-DBM groups in the main dorsal column sensory tract approaching the lesion site (p = 0.8). Thus, targeting of SnoN, a developmentally-regulated axonal transcription factor, significantly enhances axonal regeneration after adult spinal cord injury.

Assessment of in vivo axonal regeneration must ensure that regenerating axons are not mistaken for spared axons: examination of the spinal cord and medulla rostral to the lesion site showed an absence of CTB-labeled axons, indicating lesion completeness in all subjects (not shown). Taken together, these results indicate that SnoN overexpression significantly enhances axonal regeneration after spinal cord injury.

## Discussion

Findings of this study reveal for the first time that a developmentally-regulated transcription factor specifically related to extension of the developing axon, SnoN, enhances growth of adult axons both *in vitro* and *in vivo* after spinal cord injury. While the function of SnoN in neural development has been the subject of previous studies [8,9,18–21], a role for this molecule in adult nervous system injury has not previously been described.

SnoN was first identified in a screen for genes modulating the oncogene c-ski [22]. It has subsequently been shown to act as a regulator of the cellular growth state both in the context of cancer biology and neurobiology, exerting effects on a number of different cell types [23]. Many of its actions are mediated through regulation of TGF-β signaling, often interacting with Smads and components of the histone deacetylase complex to influence the cell cycle [24–26]. SnoN appears to act as an oncogene and a tumor suppressor gene [23], a role it shares with other recently identified molecules that have also been shown to modulate adult CNS axonal regeneration, including PTEN [27]. More specifically in the context of neural development, SnoN exerts effects on post-mitotic neurons by critically regulating axonal morphogenesis [8]. Knockdown of SnoN in post-natal primary granule neurons profoundly and specifically retards axon growth [8]. Levels of SnoN are dynamically regulated during development by the ubiquitin ligase Cdh1-APC, and developmental regulation of Cdh1-APC, TGFβ and SnoN levels correlates strongly with distinct periods of axonal growth and termination of axon growth [9]. More recently, roles for SnoN in neuron branching, migration and positioning have also been described [23]. With a specific role in promoting elongation of the axon, SnoN represented a compelling target for examination in a model of adult central axonal regeneration. As we now demonstrate, overexpression of SnoN indeed enhances neurite outgrowth from adult DRG neurons and enhances the regeneration of these same neurons after in vivo spinal cord injury, recapitulating its role in axonal outgrowth during development.

In general, molecular mechanisms that influence axon outgrowth during development have in many cases also been shown to influence growth of the axon after injury in adulthood, including neurotrophic factors, diffusible guidance molecules (e.g., netrins, semaphorins, ephrins), transcriptional regulators (e.g., Id2, which like SNoN is modulated by Cdh1-APC [28]) and now, SnoN. Yet the regulation of many of these molecular mechanisms during development is complex, illustrated clearly by the role of netrins and semaphorins in development [29]. Simple overexpression or knockdown approaches may not always yield clear effects. But in the case of SnoN overexpression, significantly enhanced axonal growth after spinal cord injury is observed, supporting a potentially useful translational role for this molecule in enhancing plasticity and regeneration.
